# Video-based simulations in teacher education: the role of learner characteristics as capacities for positive learning experiences and high performance

**DOI:** 10.1186/s41239-022-00351-9

**Published:** 2022-09-01

**Authors:** Michael Nickl, Sina A. Huber, Daniel Sommerhoff, Elias Codreanu, Stefan Ufer, Tina Seidel

**Affiliations:** 1 TUM School of Social Sciences and Technology, Technical University of Munich (TUM), Arcisstraße 21, 80333 Munich, Germany; 2 Mathematics Education, Leibniz Institute for Science and Mathematics Education (IPN), Kiel, Germany; 3 Chair of Mathematics Education, Ludwig-Maximilians-Universität München (LMU), Munich, Germany

**Keywords:** Assessment skills, Assessment process, Authenticity, Higher education, Latent profile analysis, Learner characteristics, Person-centered approaches, Teacher education, Video-based simulation

## Abstract

Assessing students on-the-fly is an important but challenging task for teachers. In initial teacher education, a call has been made to better prepare pre-service teachers for this complex task. Advances in technology allow this training to be done through authentic learning environments, such as video-based simulations. To understand the learning process in such simulations, it is necessary to determine how cognitive and motivational learner characteristics influence situative learning experiences, such as the perception of authenticity, cognitive load, and situational motivation, during the simulation and how they affect aspects of performance. In the present study, *N* = 150 pre-service teachers from German universities voluntarily participated in a validated online video-based simulation targeting on-the-fly student assessments. We identified three profiles of learner characteristics: one with above average knowledge, one with above average motivational-affective traits, and one with below average knowledge and motivational-affective traits. These profiles do not differ in the perception of the authenticity of the simulation. Furthermore, the results indicate that the profiled learners navigate differently through the simulation. The knowledgeable learners tended to outperform learners of the other two profiles by using more learning time for the assessment process, also resulting in higher judgment accuracy. The study highlights how learner characteristics and processes interact, which helps to better understand individual learning processes in simulations. Thus, the findings may be used as a basis for future simulation research with a focus on adaptive and individual support.

## Introduction

Assessing students on-the-fly is a key professional skill that teachers use daily (Urhahne & Wijnia, 2021). Consider a class where each student is solving a mathematical task individually. The teacher discusses the task with individual students and provides support. At the end of the lesson, the teacher should know how many students have solved the task, how they solved it, and what difficulties they encountered. This information is required to adequately plan the next lesson and to provide adaptive, individual support for each student. When taking the short length of lessons and the high number of students per class into account, it becomes clear that assessing students is a challenging task for in-service teachers. This is manifested in a quite strongly varying judgment quality of teachers (Urhahne & Wijnia, 2021). Because in-service teachers face problems with formatively assessing students, it can be assumed that this task is even more challenging—and possibly overwhelming—for pre-service teachers (Levin et al., 2009). Despite the fact that teacher educators agree that this task should be addressed from the beginning of teacher education (KMK, 2004), it is not yet clear how to best support the acquisition of these skills (Chernikova et al., 2020a, 2020b). To train future teachers in these diagnostic skills, performance-oriented and practice-based learning opportunities are required (Ferry et al., 2006). Technological advancements in education, currently explored by researchers worldwide, provide a rich resource for teacher education. From a conceptual standpoint, educational technological tools for teacher education must approximate actual teaching practice yet break down the complexity of real-world professional situations into more manageable learning units for novices, such as inexperienced pre-service teachers (Grossman et al., 2009). Video-based training tools seem to achieve these goals (Borko et al., 2008; Gaudin & Chaliès, 2015; Kang & van Es, 2019). Furthermore, learning simulations provide authentic yet digestible learning opportunities for many professional skills, especially in medical education (Beaubien & Baker, 2004; Khan et al., 2011) but also recently in teacher education (Amador, 2017). Thus, the combination of videos and simulations in digital learning environments is a promising educational technology tool for teacher education (Kramer et al., 2020).

### Research aims﻿

To understand pre-service teachers’ learning in such video-based simulations, individual learning processes need to be studied in detail with the aim to provide an empirical basis for future personalized adaptations. To explore these individual learning processes, we take relevant pre-service teachers’ cognitive and motivational-affective learner characteristics, their situative learning experiences during learning with a video-based simulation (i.e., perceptions and experiences, such as motivation and involvement), and actual learning activities into account (Heitzmann et al., 2019). According to the model developed by Heitzmann et al. (2019), we assume that learner characteristics have an impact on the way situative learning experiences are perceived and on the depth of the learning activities carried out in the video-based simulation. In turn, this interplay affects learning outcomes, such as the quality of final performance (Deci & Ryan, 2000; Froiland & Worrell, 2016).

For the long-term perspective of designing adaptive video-based simulations and providing personalized instructional support, it is an important first step to identify specific learner needs based on relevant learner characteristics (Plass & Pawar, 2020; Tetzlaff et al., 2021). To identify these needs, we suggest (1) using a person-centered approach and identifying learner characteristic profiles and (2) analyzing the possible needs of each profile (e.g., by exploring how these learner characteristic profiles experience learning during the simulation). To our knowledge, profiles of pre-service teachers’ learner characteristics in video-based simulations and how these profiles affect processes and skills to be learned in video-based simulations have not yet been reported in the literature.

Regarding the present study, our team developed a video-based simulation that can serve as a learning environment to sharpen pre-service mathematics teachers’ formative assessment skills regarding student mathematical argumentation. By using the simulation, we aimed to promote the understanding of learners’ situative learning experiences and performance in simulations depending on their individual learner characteristics. The findings of the study can serve as a basis for further adaptive personalizations of simulation-based learning. In detail, we investigated the extent to which distinct learner characteristic profiles can be identified and whether profiles can explain differences between learners regarding their situative learning experiences and learning activities during the simulation. Furthermore, we explored differences between learner profiles with regard to achieved learning outcomes. Using a person-centered approach in combination with pre-service teacher training in a practice-oriented simulation represents a novel approach in current research. The findings of this study contribute to advancing the field of educational technologies and simulations in the field of teaching, which is highly complex and practice-oriented. Furthermore, the methodological approach of person-centered analyses can inform researchers in the broader field of educational technologies regarding ways to consider complex learner characteristics as prerequisites for learning. Overall, we assume that empirical studies contribute to a better understanding of how to best design educational technologies for teacher education and to adapt them to the personal needs of pre-service teachers as learners.

#### Educational technologies in teacher education: video-based simulations

Novice teachers often experience difficulties in the initial years of practicing teaching (Correa et al., 2015; Dicke et al., 2016). Many are overwhelmed by the complex realities of everyday classrooms and experience what is known as “practice shock” (Stokking et al., 2003); however, Stokking et al. (2003) point out that “the extent of such difficulties depends on the training they have received” (p. 331). Therefore, the ways in which teacher education provides opportunities to apply knowledge in practice situations is of high importance. In most cases, teacher education programs focus on lecture-style knowledge acquisition paired with practice periods or internships (Musset, 2010); however, this approach does not seem sufficient to appropriately prepare pre-service teachers (Levin et al., 2009). Thus, we examine alternative learning settings in this initial phase of pre-service teacher learning (Ferry et al., 2006; Stokking et al., 2003). As a conceptual foundation for the design of such learning settings, Grossman et al. (2009) highlight that (1) teacher educators should strive for approximations of practice that provide an authentic setting for relevant learning to take place and that (2) such environments should be reduced in complexity in the form of systematic decompositions of practice.

Incorporating videos into teacher education and professional development can ensure that both of these criteria are met (Borko et al., 2008; Codreanu et al., 2020; Santagata, 2009). Although which learning environment and which learner characteristics increase the perception of authenticity has yet to be determined by research (Betz, 2018; Betz et al., 2016; Gulikers et al., 2005; Mikeska & Howell, 2021), videos have been proven reliable in preserving the authenticity of snippets of everyday teaching and learning (Gaudin & Chaliès, 2015; Kang & van Es, 2019; Tekkumru-Kisa & Stein, 2017). At the same time, the practice of selecting distinct situations, segmenting them into meaningful learning units, and highlighting particularly relevant situations in staged videos offers many technical opportunities for a sufficient decomposition of complex classroom realities (Derry et al., 2014; Thiel et al., 2020). Given these positive uses of video in teacher education, it is not surprising that video-based tools and environments have been shown to be quite effective in teacher education (Gaudin & Chaliès, 2015). Stürmer et al. (2013), for instance, compared different teacher education course formats and showed that the highest gains in teachers’ knowledge and skills were reached during a video-based course.

The effectiveness of knowledge acquisition when using video can be further improved when videos are embedded in more interactive, individualized, and adaptive formats, such as video-based simulations (Chernikova et al., 2020a, 2020b; Derry et al., 2014; Plass & Pawar, 2020). Through a simulation of a particular practice situation represented via a digital environment with embedded video clips, pre-service teachers can not only experience aspects of authentic and relevant teaching tasks within a complexity-reduced setting but can also actively participate in and interact with such tasks (Gredler, 2004). As mentioned, this type of technology-based environment can also be individualized. Adaptation can be based on learner characteristics, such as cognitive and motivational-affective dispositions (Heitzmann et al., 2019; Plass & Pawar, 2020).

#### Relevant learner characteristics

Just as teachers need to adapt their teaching to their students’ individual needs, teacher education must also consider what individual teachers “bring to learning” (Laurillard, 2002, p. 25). Some cognitive and motivational-affective dispositions are viewed as highly relevant prerequisites for teacher learning (Blömeke et al., 2015; Heitzmann et al., 2019).

*Cognitive characteristics.* Important cognitive capacities in teacher education are related to the acquisition of professional knowledge, particularly in the area of content knowledge (CK) and pedagogical content knowledge (PCK; Ball et al., 2008; Baumert et al., 2010; Shulman, 1986). During the last decade, ample evidence has been gathered regarding the structure of professional knowledge in the teaching domains and with regard to the relationship between professional knowledge and professional performance. Professional knowledge has been shown to be highly predictive of instructional quality as well as student learning outcomes (Baumert et al., 2010; Kunter et al., 2011, 2013). Focusing on the role of professional knowledge in teachers’ professional performance in assessing students, the empirical findings are mixed. Some studies have shown that experienced teachers can properly engage in assessment activities once they have acquired appropriate CK (Aschbacher & Alonzo, 2006). Other studies comparing primary and secondary teachers’ assessment skills have indicated that CK does not play a major role (Karing, 2009). Regarding the role of PCK in the application of assessment skills, studies have shown mixed results, ranging from weak correlations between PCK and judgment accuracy (McElvany et al., 2009) to large effects from PCK supportive instruction (Ostermann et al., 2018).

*Motivational-affective characteristics.* Motivational dispositions, such as interest in teaching and learning, self-efficacy, and self-regulation,^1^ represent a further set of relevant learner characteristics (Dicke et al., 2016; Holzberger et al., 2021; Mattern & Bauer, 2014; Toropova et al., 2021), particularly with regard to teachers’ roles in successfully handling assessment situations (Heitzmann et al., 2019). Self-regulation skills have been shown to be highly relevant for learning in many educational contexts and are also expected to play an important role in pre-service teachers’ acquisition of complex skills (Dent & Koenka, 2016; Kara et al., 2021). Self-efficacy has also repeatedly been shown to be an important capacity for learning in general (Dupeyrat et al., 2011; Praetorius et al., 2016). Some studies have also highlighted its relevance to teaching (Tschannen-Moran et al., 1998); however, other studies, such as Klug et al.’s (2016) study, have shown no relevant relationship between self-efficacy and teachers’ assessment skills. Likewise, interest in teaching and learning has been shown to play an important role in high-quality teaching in general. At the same time, studies have also shown positive relationships between teacher interest and professional learning success (Ainley et al., 2002; Pancsofar & Petroff, 2013; Schiefele et al., 2013).

While current evidence on pre-service teachers’ relevant learner characteristics is still sparse, it seems plausible to apply the most established cognitive and motivational-affective dispositions in teacher research to the learning setting of video-based simulations and to the context of formatively assessing student skills. Thus far, studies indicate that participants with varying levels of prior professional knowledge and motivational-affective characteristics interact differently with video-based learning environments; consequently, they might also benefit from video-based simulations in varying ways (Blomberg et al., 2011; ChanLin, 2001). Situative learning experiences as well as assessment skills as actual learning activities and learning outcomes could serve as helpful indicators to trace these different interactions in video-based simulations, which allows for further clarifying the potential benefits of these educational technologies in teacher education.

#### Situative learning experiences during video-based simulations

To clarify the potential benefits of video-based simulations for teacher education, the learning processes during an engagement with a simulation can provide important clues (Derry et al., 2014; Moreno & Mayer, 2007). Based on indicators that are relevant to stimulating motivation and deep-learning activities (Heitzmann et al., 2019), differences in individual learning outcomes can be further clarified. Thus, studying the interplay of relevant learner characteristics, learner experiences during a simulation, and resulting performance (as learning outcomes) provides valuable evidence to further improve simulations through instructional support (e.g., by adding personalized scaffolds and feedback).

Regarding situative learning experiences, balancing authenticity and involvement with an appropriate level of cognitive demand as well as motivational-affective stimulation is considered one of the main challenges when developing a video-based simulation (Codreanu et al., 2020; Derry et al., 2014; Grossman et al., 2009).

*Cognitive demand.* In the field of research on learning complex skills, various cognitive design criteria are discussed. Among didactic-related design principles (Achtenhagen, 2001), research on learning with media has pointed to different aspects of the learning process that can distract the learner from the actual learning activity (Mayer, 2014). In this regard, cognitive load theory is well-established and proposes the reduction of design-related, *extraneous,* and task-related *intrinsic* cognitive load to allow for an increase in learning-related, *germane* cognitive load (Sweller et al., 1998). Particularly in the context of technology-enhanced media, such as video-based simulations, researchers often draw on this classification, indicating higher learning outcomes when learning environments do not overwhelm learners and the task is well-structured (e.g., by segmenting video clips; Lange & Costley, 2020; Sweller et al., 2019).

*Motivational-affective stimulation.* Learner experiences in video-based simulations are not only determined by their cognitive demand but also by motivational and affective stimulation from the environment. Video examples and learning tasks in video-based simulations represent stimuli that affect motivation. If simulations are experienced as personally relevant (e.g., due to personal or professional interests) or easy (e.g., due to high self-efficacy), the subjective value or the expectancy component of motivation for engaging in learning activities is increased, respectively (see Wigfield & Eccles, 2000), which has been shown to positively affect performance and learning outcomes (Chen & Wu, 2012; Froiland & Worrell, 2016).

*Presence.* Regarding simulations, the experience of presence through authenticity and involvement plays a key role and positively affects performance and learning outcomes (Grossman et al., 2009; Schubert et al., 2001). Prior research on presence has shown positive effects on students’ learning outcomes (Chernikova, et al., 2020a, 2020b; Mikeska & Howell, 2021; Stevens & Kincaid, 2015). In particular, little research has been conducted on understanding learner characteristics that might serve as more or less favorable capacities for experiencing a digital learning environment as authentic. Betz et al. (2016) found personal factors, such as prior knowledge and interest, to be relevant, whereas previous empirical studies have shown mixed findings (Betz, 2018; Chernikova, et al., 2020a, 2020b; Gulikers et al., 2005). Thus, it is still rather unclear which personal and situational characteristics might lead to more positive perceptions of authenticity.

#### Assessment skills as learning activities and learning outcomes

Formative assessment skills of teachers are considered fundamental professional skills of the teaching profession and are highly relevant for teaching and learning success (Herppich et al., 2018; Südkamp et al., 2012; Weinert et al., 1990). Teachers also consider a formative assessment an “integral part of the teaching–learning process” (Barnes et al., 2015, p. 293). This attention is mirrored in research, where a growing conceptualization of assessment competences differentiates between professional knowledge (as outlined), assessment activities *during* an assessment process, and *final* judgment performance, such as reaching a high judgment accuracy (Blömeke et al., 2015; Glogger-Frey et al., 2018; Heitzmann et al., 2019).

*Assessment process.* To explain differences in judgments and their quality, recent research has increasingly focused on the assessment process (Glogger-Frey et al., 2018; Herppich et al., 2018; Kramer et al., 2021; Urhahne & Wijnia, 2021). One promising approach to understanding important processes during an assessment and their effects on judgment quality is taking the notes of the assessors into account (Codreanu et al., 2021).

*Judgment quality.* The appropriateness of an assessment is reflected in its final judgment quality (Artelt & Gräsel, 2009). A prominent measure for rating the quality of a judgment is its accuracy (Chernikova et al., 2020a, 2020b). Regarding teacher–student interactions with few students, this judgment accuracy is often regarded along the dimensions of comparing students with each other and rating students’ characteristics compared to fixed categories (Schrader & Helmke, 1987). While in-service as well as pre-service teachers tend to perform quite well in ranking students, research points toward difficulties in accurately determining individual students’ knowledge levels and skills (Südkamp et al., 2008, 2012; Urhahne & Wijnia, 2021).

## Research questions

The aim of this study was to examine the role of relevant learner characteristics for situative learning experiences and performance in a video-based simulation in teacher education. The following research questions were addressed:

1. Do participants’ relevant learner characteristics show distinct learner profiles? If this is the case, which learner characteristic profiles can be identified for pre-service teachers?
2. Do learner characteristic profiles affect how pre-service teachers experience their engagement with the simulation (motivation, presence, cognitive load)?
3. Do learner characteristic profiles affect pre-service teachers’ assessment skills with regard to the assessment process (watching videos, taking notes) as well as their final judgment (judgment report, judgment accuracy)?

We conjectured that participants’ response patterns for cognitive and motivational-affective learner characteristics would be represented through a profile of comparably higher or lower cognitive and motivational-affective dispositions. In addition, we assumed a profile of comparably high motivation but lower professional knowledge and learners with a profile of comparably high knowledge but average to lower motivation (RQ1).

Furthermore, we assumed that learners with a high-motivation profile would be predisposed to experience more presence during the video-based simulation and would be more situatively motivated. For cognitive demand, we assumed that learners with a high-knowledge profile would also experience a more appropriate cognitive demand, such as with regard to reporting a comparably high germane load and a low extraneous load. We assumed that learners with both low cognitive and low motivational-affective dispositions would be at a disadvantage for positive learning experiences during the simulation (RQ2).

Similar assumptions were made with regard to learning outcomes. We expected high cognitive dispositions and high motivational-affective dispositions to positively affect assessment skills, and we thus expected learners with both dispositions low (high) to have the lowest (highest) quality of assessment process as well as final judgment accuracy. With regard to the two profiles with either high motivational-affective or high cognitive dispositions, we expected high motivation to partially compensate for a lack of knowledge and vice versa, leading to a similar quality of the assessment process and of the final judgment (RQ3).

## Methods

### Sample

During the middle of regular university courses in 2020 and 2021, pre-service teachers enrolled in seven German university teacher education programs were invited for voluntary participation from the course instructor via an online learning management system. Participation was remunerated but did not influence course grading or students passing. A total of *N* = 150 pre-service teachers participated in the study [101 f (67.3%), 47 m (31.3%), 2 NA (1.3%), M_age_ = 23.1y (*SD* = 3.4y)].^2^ Most participants were in the middle of their studies (M_semester_ = 5.0, *SD* = 2.5). All participants provided informed consent. The study as well as the storage and processing of the data were approved by the German Psychological Society (DGPs) and TUM data protection office.

### Design of the study and video-based simulation

The study was conducted online and contained the video-based simulation as well as measures for relevant learner characteristics and learning experiences during the simulation. First, participants were asked to complete a questionnaire and test regarding their cognitive (knowledge) and motivational-affective dispositions. During and shortly after the video-based simulation, participants answered questions about their situative learning experiences during the simulation. In the video-based simulation (for a detailed description, see Codreanu et al., 2020), participants assumed the role of an observing pre-service intern in a simulated classroom. Their task was to assess the mathematical argumentation skills of two simulated students by observing typical one-on-one teacher–student interactions during class. These interactions were presented in short video clips. Each video clip included the simulated teacher and one of two simulated students from seventh grade at a German high school. Each clip lasted approximately one minute. In these video clips, the simulated teacher and the simulated student discuss the student’s progress and argumentation in the context of a geometry proof the student is completing. Participants were asked to take notes during these videos. In the course of the simulation, participants could choose to watch multiple video clips of each simulated student (at most eight per simulated student and 10 in total) before providing their final judgment. In addition, they were encouraged not to watch more video clips than necessary to reach an assessment decision. After watching the videos, participants’ judgment accuracy was measured based on their judgment of the simulated students’ levels of mathematical argumentation skills. The assessment was based on three content dimensions (mathematical content knowledge, methodological knowledge, and problem-solving strategies) derived from Reiss and Ufer (2009), for which behavioral cues were disseminated throughout the video clips (Codreanu et al., 2020).

### Measures and instruments

#### Learner characteristics

We measured the motivational-affective and cognitive dispositions of the pre-service teachers as learner characteristics. We also measured their professional knowledge, individual interest, self-efficacy, and perceived self-regulation abilities.

*Professional knowledge.* The assessment of the pre-service teachers’ professional knowledge contained scales for CK and PCK and was based on the widely accepted considerations by Shulman (1986), see also Förtsch et al. (2018) in the context of assessment skills. The CK scale focused on knowledge matching the mathematical contents of the simulated assessment situation. The following is an example item: “Mark all statements as true or false: (a) There are rhombi that are squares; (b) each parallelogram is a kite; (c) there are rhombi that are kites; (d) each rhombus is a trapezoid.”

The PCK scale focused on the educational potential of geometry tasks and teachers’ responses to students’ statements related to these kinds of tasks (Ball et al., 2008). The following is an example item: “The following task is given: In a trapezium ABCD is AB || CD. Further, α = 31°, $\upgamma$=78°. Calculate β and δ! What learning objective(s) can you pursue with this task? Students can (a) apply congruence theorems, (b) name characteristics of trapezia, (c) use interior angle sum of quadrilaterals, (d) use angle theorems for intersected parallels.”

Both scales consisted of eight items each, which were developed by the research team to fit the content of the video-based simulation. Items of the PCK scale were true–false with four possible answers. The CK scale had five true–false items and three open-text items.

*Individual interest.* Individual interest items focused on participants’ interest in aspects relevant to the simulation (student assessment, geometry, mathematics education). Each aspect was measured with three items adapted from Rotgans and Schmidt (2014). Items were rated on a five-point Likert scale. Cronbach’s α was larger than 0.86 for each of the three subscales. The following is an example item: “I find mathematics education interesting.”

*Self-efficacy*. Self-efficacy was measured with six items stemming from PISA (Kunter et al., 2002). The items measured whether participants felt confident about their assessment skills and whether they felt able to solve assessment situations (Cronbach’s α = 0.90). The items were rated on a five-point Likert scale. The following is an example item: “If I am to solve difficult assessment cases, I believe that I can do it.”

*Self-regulation.* Trait-like self-regulation skills were measured using six items on a four-point Likert scale. The items were adapted from Kunter et al. (2016) (Cronbach’s α = 0.83). The following is an example item: “I really do my best to achieve my goals.”

#### Situative learning experiences during the simulation

*Motivation*. Situative motivation was captured by two scales following the expectancy-value theory of Wigfield and Eccles (2000), one focusing on participants’ subjective task value with four items on a five-point Likert scale (Wigfield, 1994) and one focusing on their expectancy to succeed with four items on a seven-point Likert scale (Rheinberg et al., 2001) (task value: Cronbach’s α = 0.83; expectancy: Cronbach’s α = 0.80). The following are example items: “It is useful to deal with this task” (task value) and “I think everybody is able to solve this task” (expectancy).

*Presence.* Four items regarding participants’ *cognitive involvement* were derived from Seidel et al. (2011), Frank (2015), and Vorderer et al. (2004) for the study. These items were rated on a five-point Likert scale (Cronbach’s α = 0.71). The following is an example item: “I focused heavily on the situation.” For *authenticity*, we used six items that were adapted from Seidel et al. (2010) and Schubert et al. (2001), which were rated on a five-point Likert scale (Cronbach’s α = 0.87). The following is an example item: “I think the simulation is authentic.”

*Cognitive load*. To measure the perceived cognitive demand, we implemented a scale on cognitive load. This scale was adapted from Klepsch et al. (2017) and consisted of three subscales regarding extraneous cognitive load (three items), intrinsic cognitive load (two items), and germane cognitive load (two items). These items were rated on a seven-point Likert scale. The following are example items: “In this task, it is laborious to find the important information” (extraneous load), “The task is very complex” (intrinsic load), and “When doing the simulation, it was important to me to get everything right” (germane load).

#### Assessment skills

Based on the conceptualization of assessment skills from Heitzmann et al. (2019), we considered the assessment process as well as the final judgment when investigating participants’ assessment skills. As measures from the process, we took the number of videos participants watched and the number of words the participants wrote in their *notes* into account. For the final judgment, we studied the number of words participants wrote in their *final judgment* and the participants’ judgment accuracy. Judgment accuracy was measured as follows. Participants rated each simulated student’s mathematical argumentation skills concerning three different content dimensions—mathematical content knowledge (Weigand et al., 2014), methodological knowledge (Heinze & Reiss, 2003), and problem-solving strategies (Schoenfeld, 1992). For each content dimension, participants answered two or three items on a four-point Likert scale (a total of eight items per simulated student). Each item addressed a distinct aspect of the simulated students’ mathematical argumentation skills in terms of geometry proofs. Participants’ judgments were scored against an expert solution developed by two experts from mathematics education (expert interrater agreement Cohen’s κ = 0.80). Agreement with the expert rating was scored with one point (expert match); otherwise, participants received zero points (no expert match). Participants’ judgment accuracy was then calculated as the sum of all rating items (three for mathematical content knowledge, three for methodological knowledge, and two for the problem-solving strategies for each of the two students). Thus, participants’ judgment accuracy scores could range from 0 to 16 points.

#### Data analysis

Personal data needed for the remuneration were stored separately from the anonymized data used for analysis. To provide better comparability across variables, all response scales regarding learner characteristics and situative learning experiences were z-standardized. This is also beneficial for the convergence of the latent profile analysis (LPA), which was run subsequently to answer RQ1. LPA is a statistical procedure that is applied to identify underlying class membership among participants. LPA was run with Mplus (Muthén & Muthén, 1998–2017) using the professional knowledge scales (CK, PCK) and the three motivational scales (individual interest, self-efficacy, and self-regulation) as indicator variables for learner characteristic profiles. We tested models between one and five profiles. The best-fitting latent profile model was determined by examining relevant fit indices (AIC, BIC, adjusted BIC, Entropy, and LRT and BLRT *p*-values) as well as considering content-related interpretability (Ferguson et al., 2020; Pastor et al., 2007; Schnitzler et al., 2020).

To answer RQ2 and RQ3, we calculated the means of the analyzed variables for each of the identified profiles. We used a widely accepted three-step approach: first, estimating model fit; second, assigning participants to one class; and third, evaluating these assignments with respect to a model (Dziak et al., 2016). As Bolck et al. (2004) outlined, simply assigning participants, for example, based on their most probable class membership (modal assignment) or softly assigning participants by their class membership probability (proportional assignment) leads to a statistical error. Therefore, we used the modified BCH correction method (Vermunt, 2010). In the BCH method, weights are calculated based on the probabilities of assigning participants to the wrong group (Huang et al., 2017). We then calculated the weighted mean and standard error for each profile and for each of the situative and outcome variables. To investigate the statistical significance of the differences between the profiles, we obtained ${\chi }^{2}$ and corresponding *p*-values from a Wald test (Spurk et al., 2020), which are reported for each investigated scale.

## Results

### Descriptive analysis

Descriptive statistics of all learner characteristics used for the LPA are presented in Table 1. To enhance comparability, variables are presented on a scale from 0 to 1.

**Table 1:** Descriptive statistics for LPA indicator variables

	CK	PCK	Interest	Self-efficacy	Self-regulation
*M (SD)*	0.67 (0.17)	0.66 (0.10)	0.75 (0.16)	0.62 (0.17)	0.79 (0.16)
Min	0.29	0.31	0.25	0.08	0.33
Max	1.00	0.91	1.00	1.00	1.00

Scales from 0, theoretical minimum, to 1, theoretical maximum

### Identification and description of latent trait-profiles

**Fig. 1:**
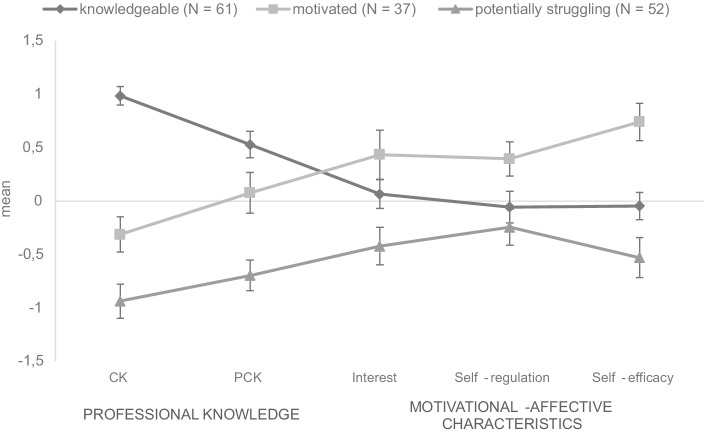
Mean values and standard deviations of the three profiles regarding z-standardized LPA indicators

For the following analyses, we used z-standardized values. To answer RQ1, the LPA method was applied. Three distinct profiles were identified based on pre-service teachers’ cognitive and motivational-affective learner characteristics. We based the decision for selecting the three-profile solution on relevant LPA criteria (AIC, BIC, adjusted BIC, entropy, LRP, and BLRT *p*-values). The detailed values for these criteria can be found in Table 2. AIC, BIC, and adjusted BIC indicators do not change substantially for solutions with two or more profiles. Entropy slightly increases for the four-profile solution compared to the three-profile solution. The LRT *p*-value shows that switching from a three-profile solution to a four-profile solution does not significantly improve model fit. Because the BLRT *p*-value suggests a significant improvement in the model fit when comparing the two-profile to the three-profile solution, we assume the latent three-profile solution shows the best fit. Furthermore, average posterior probabilities for each profile of the three-profile-solution contain maximum values on the diagonal, indicating a good distinction between profiles, as shown in Table 3. The mean values of the three profiles are visualized in Fig. 1.

**Table 2:** Statistical criteria of different profile solutions

Number of profiles	AIC	BIC	aBIC	Entropy	LRT *p*-value	BLRT *p*-value
1	3858.10	3912.30	3855.33			
2	2106.31	2154.48	2103.84	0.73	0.052	< 0.001
**3**	**2092.08**	**2158.31**	**2088.69**	**0.71**	**0.052**	**< 0.001**
4	2085.45	2169.75	2081.13	0.80	0.393	0.077
5	2080.55	2182.91	2075.30	0.78	0.088	0.050

Chosen solution is highlighted in bold

**Table 3:** Average probabilities for most likely latent profile membership (row) by latent profile (column)

	1	2	3
1	**0.87**	0.11	0.02
2	0.12	**0.80**	0.09
3	0.01	0.07	**0.92**

Highest row values are highlighted in bold

*Knowledgeable profile*. Learners of the first profile, labeled “*knowledgeable*,” show above-average prior knowledge as well as medium values regarding interest, self-efficacy, and self-regulation. When assigning pre-service teachers to a group based on their most likely class membership, this group contains *n* = 61 pre-service teachers (40.7%).

*Motivated profile*. In the second profile, labeled “*motivated*,” there are contrasting results: pre-service teachers in this profile showed high motivational-affective learner characteristics but a rather average performance regarding prior knowledge (CK rather below average, PCK slightly above average); *n* = 37 pre-service teachers were assigned to this group (24.7%).

*Potentially struggling profile.* The third profile (*n* = 52 pre-service teachers, 34.7%) is called “*potentially struggling*” because their learner characteristics are below average regarding all abovementioned facets and are below the other two profiles.

### Situative learning experiences during the simulation

**Fig. 2:**
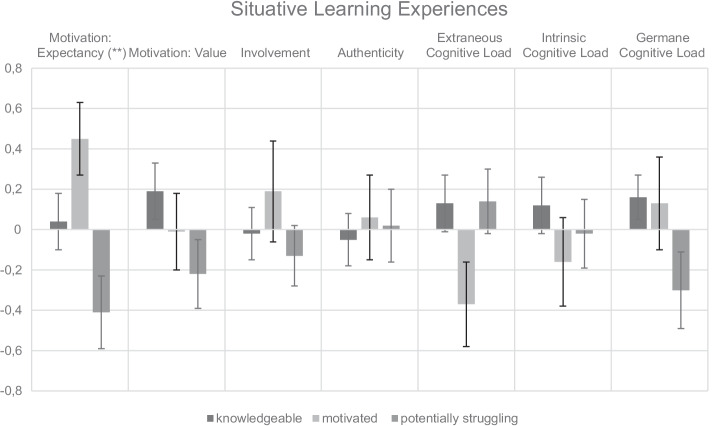
Mean values and standard error bars of the three profiles regarding situative learning experiences

To answer RQ2, we compared the three latent trait-profiles with respect to participants’ situative learning experiences during the simulation using the BCH method (see Fig. 2 and Table 4).

**Table 4:** Mean, standard error, and p-value of overall group comparisons regarding learning experiences and assessment skills

	*p*-value	Knowledgeable *M (SE)*	Motivated *M (SE)*	Potentially struggling *M (SE)*
Situative learning experiences				
Motivation: expectancy	**0.008**	0.04 (0.14)	0.45 (0.18)	− 0.41 (0.18)
Motivation: value	0.168	0.19 (0.14)	− 0.01 (0.19)	− 0.22 (0.17)
Involvement	0.624	− 0.02 (0.13)	0.19 (0.25)	− 0.13 (0.15)
Authenticity	0.894	− 0.05 (0.13)	0.06 (0.21)	0.02 (0.18)
Extraneous cognitive load	0.165	0.13 (0.14)	− 0.37 (0.21)	0.14 (0.16)
Intrinsic cognitive load	0.572	0.12 (0.14)	− 0.16 (0.22)	− 0.02 (0.17)
Germane cognitive load	0.106	0.16 (0.11)	0.13 (0.23)	− 0.30 (0.19)
Assessment skills				
Number of videos	**0.028**	7.13 (0.35)	6.01 (0.58)	5.75 (0.44)
Number of words: notes	0.186	128.96 (10.65)	106.05 (14.66)	101.35 (12.5)
Number of words: judgment	0.124	129.60 (11.82)	131.47 (17.68)	100.14 (10.36)
Judgment accuracy	0.116	5.95 (0.36)	5.26 (0.50)	4.86 (0.40)

Significant differences between profiles are highlighted in bold

*Motivation.* Significant group differences were found for the expectancy to accomplish the diagnostic task but not for the perceived subjective value of the task. Thereby, the *potentially struggling* pre-service teachers’ expectancy was significantly lower in comparison to the expectancy of the *motivated* (${\chi }^{2}$ = 9.51, *p* = 0.002) and *knowledgeable* pre-service teachers (${\chi }^{2}$ = 4.12, *p* = 0.043). The *motivated* and *knowledgeable* participants did not significantly differ with regard to expectancy (${\chi }^{2}$ = 2.76, *p* = 0.097). With regard to task value, the descriptive results suggested the following descending rank order: *knowledgeable*, *motivated*, and *potentially struggling*.

*Presence.* For involvement, group differences regarding motivation did not result in significant differences in perceived involvement. Descriptively, the *motivated* profile showed the highest values, whereas the *potentially struggling* profile showed the lowest values. Regarding authenticity, mean differences were smaller, yielding no significant differences.

*Cognitive load.* No significant overall effects of profiles were found. On a descriptive basis, *motivated* pre-service teachers perceived the least extraneous and intrinsic cognitive load. Regarding the germane cognitive load, *potentially struggling* teachers perceived substantially less of this kind of load, especially significantly less than the *knowledgeable* profile (${\chi }^{2}$ = 4.47, *p* = 0.034). The *motivated* and the *knowledgeable* profile did not differ substantially (${\chi }^{2}$ = 0.01, *p* = 0.924).

### Assessment skills as learning outcome indicators

**Fig. 3:**
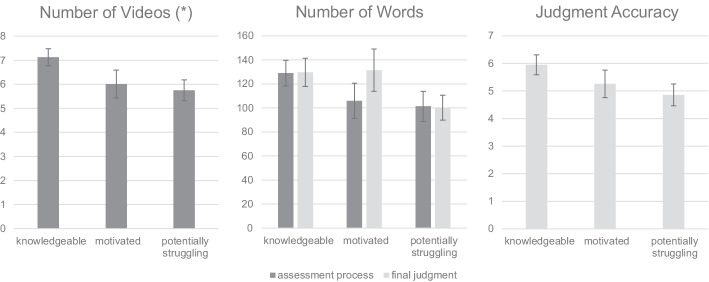
Profiles’ means and standard errors regarding the assessment process (dark gray) and final judgment (light gray)

Regarding RQ3, we compared the three profiles between measures indicating an effective assessment process and those indicating an effective final judgment (Fig. 3; Table 4, lower part).

*Assessment process.* Whereas the mean number of words written in the notes did not reveal significant overall differences between profiles, a significant overall effect was found with regard to the number of videos watched (${\chi }^{2}$ = 7.12, *p* = 0.028). For both process measures, the descending rank order was the same: *knowledgeable*, *motivated*, and *potentially struggling*. The difference between *knowledgeable* and *potentially struggling* pre-service teachers reached significance regarding the number of videos watched (${\chi }^{2}$ = 6.06, *p* = 0.014). All other differences were not significant.

*Judgment*. Regarding the final judgment, no significant overall differences between profiles were found. Descriptively, a descending order in judgment accuracy with regard to *knowledgeable*, *motivated*, and *potentially struggling* was indicated, mirroring the rank order from the assessment process. Comparing the judgment accuracy of the *knowledgeable* with the *potentially struggling* profile, a significant difference was found (${\chi }^{2}$ = 4.13, *p* = 0.042). For words written in the judgment, one change in comparison to the process rank order occurred: *motivated* pre-service teachers wrote slightly more words than *knowledgeable* ones.

## Discussion

Simulations offer authentic learning opportunities (Codreanu et al., 2021; Kramer et al., 2020) that have been proven to facilitate the highly complex yet important assessment skills already in pre-service teacher education at university (Chernikova, et al., 2020a, 2020b; Levin et al., 2009; Urhahne & Wijnia, 2021); however, to better understand individual learning processes in such simulations, it is important to consider the triad of individual learner characteristics, situative learning experiences, and outcomes (Heitzmann et al., 2019).

### Individual learner characteristics (RQ1)

To examine the role of individual learner characteristics, person-centered approaches are needed (Tetzlaff et al., 2021). In this study, it was possible to identify different learner profiles. Our analyses revealed three learner characteristics profiles: two inconsistent profiles with (1) above-average knowledge but average motivation-affect (*knowledgeable* profile), and (2) high motivation-affect but average to low knowledge (*motivated* profile). In addition, one consistent profile was identified with below-average knowledge and motivational-affective characteristics (*potentially struggling* profile). This partially confirms our hypotheses: we indeed identified two profiles with high cognitive and rather average motivational-affective learner characteristics and vice versa as well as a profile below average regarding both facets; however, we did not find a profile with above average cognitive and motivational-affective learner characteristics. This might be explained by our sample of pre-service teachers being in the middle of their study programs. They might not have acquired sufficient knowledge, and if they did, they might have less experience in the classroom to be sufficiently optimistic and interested regarding the task of assessing students. Thus, a profile of high cognitive and high motivational-affective learner characteristics might indeed not be typical at this early stage of teacher education.

### The role of individual learner characteristics for situative learning experiences (RQ2)

Regarding the three learner characteristic profiles, the theoretical framework of Heitzmann et al. (2019) indicates that they influence learners’ situative learning experiences. The comparison of situative learning experiences based on the profiles revealed the highest values for the *motivated* profile regarding the expectancy component of motivation and the unproductive (i.e., extraneous and intrinsic) cognitive load. The *knowledgeable* profile perceived the highest subjective task value as a component of situational motivation. Regarding the learning-relevant germane cognitive load, the *knowledgeable* and *motivated* profiles were on a comparable and positive level, whereas the *potentially struggling* profile was below both profiles. Significant were the overall differences regarding the expectancy component and the germane-load-comparison of the *knowledgeable* and the *potentially struggling* profiles. These significant results are in line with our hypotheses that the *motivated* profile has more positive motivational-affective situative experiences (Wigfield & Eccles, 2000) and that the *knowledgeable* profile experiences more appropriate cognitive demands (Lange & Costley, 2020; Sweller et al., 2019).

Compared to these other aspects of situative learning experiences, authenticity showed only minimal descriptive differences between the profiles. Our results indicate that all participants, on average and independent of their learner characteristics, perceived the simulation as authentic. No profile had specific advantages or disadvantages in the perception of authenticity compared to the other profiles. Thus, our findings indicate that video-based simulations are generally perceived as an authentic learning environment by pre-service teachers (Codreanu et al., 2020), or as Grossman et al. (2009) point out, as an appropriate approximation of practice. This is good news, as the results indicate that instructors in pre-service teacher education can use simulations as approximations of practice without much concern about differential effects regarding the perception of authenticity based on different learner prerequisites.

### Individual learner characteristics and assessment skills (RQ3)

With respect to assessment skills, the findings of the present study tentatively point to advantages of the *knowledgeable* profile compared to both other profiles, which was indicated by a higher number of videos watched and words written in the notes as well as higher judgment accuracy. In terms of the number of words written in the judgment, the *knowledgeable* and the *motivated* profile were on par. Significant differences were found in the number of videos watched and between the *knowledgeable* and *potentially struggling* profile in judgment accuracy. These results suggest that learners with the *knowledgeable* profile used the most learning time for the assessment process by writing notes and watching videos. Therefore, learners with a *knowledgeable* profile showed more intense learning activities compared to *potentially struggling* learners, resulting in higher judgment accuracy, which was the main learning outcome of our video simulation. This also confirms our hypothesis that the assessment task is more challenging for the profile with potentially insufficient cognitive and motivational-affective learner characteristics. Surprisingly, the knowledgeable profile has advantages over the motivated profile regarding the assessment process and judgment accuracy.

One especially interesting finding in this regard is that all learners except those in the *motivated* profile wrote as many words in their ongoing notes as in their final judgment reports. Those in the *motivated* profile stand out because they wrote more words in the final judgment report than in their ongoing notes (Fig. 3, middle). To interpret this finding, we might assume that the learners in the *motivated* profile had such a high expectancy of successfully solving the tasks in the simulation that they underestimated the importance of applying deep-learning activities in the assessment process by closely watching video clips and taking meaningful notes regarding their observations. Thus, their high motivational-affective traits only manifested in the final judgment report, and this may have led to a decreased judgment accuracy compared to *knowledgeable* learners.

This emphasizes the importance of the assessment process as reflected in current teacher research (Codreanu et al., 2021; Herppich et al., 2018; Kramer et al., 2021; Urhahne & Wijnia, 2021). According to Herppich et al. (2018), differences in the judgment accuracy of teachers can be explained by qualitatively different ways in which they carry out the assessment process (e.g., in the way they notice and process information, form hypotheses, or collect information). Differences in learner characteristics can affect all these processes, leading, for example, those in the *motivated* profile to carry out relevant learning activities in the assessment process in a somewhat more superficial way (Praetorius et al., 2016). The lack of knowledge and motivation of those in the *potentially struggling* profile should also become evident during the assessment process (Stürmer et al., 2015). Thus, an individual adaptation of the simulation might provide further support for learners with diverse capacities (Plass & Pawar, 2020; Tetzlaff et al., 2021). For the *motivated* profile, conceptual scaffolding, such as prompts, which give greater guidance through the assessment process, could be helpful. This type of support may also be useful for *potentially struggling* learners (Chernikova et al., 2020a, 2020b). In addition, utility value interventions may also support performance for learners with low motivation (Hulleman & Harackiewicz, 2021; Wigfield & Eccles, 2000).

### Learner characteristic profiles and ways of navigating the simulation

Combining results regarding all research questions, the *knowledgeable* profile seems to navigate quite effectively through the simulation, including also rather positive learning experiences. Conversely, the *potentially struggling* profile navigated least effectively through the simulation and had rather negative learning experiences. The *motivated* profile as the third identified learner profile, which is characterized by a comparably high expectancy component in motivation, proved to be particularly interesting in the interplay of prerequisites, learning experiences, and assessment skills.

The *motivated* profile showed quite positive learning experiences, such as the highest optimism for solving the assessment task properly, that is, to assess the two students in the simulation adequately; however, when examining the assessment process and judgment accuracy, the *motivated* profile navigated slightly less effectively through the simulation than the *knowledgeable* profile, indicating that particularly high motivation does not compensate for less professional knowledge compared to the *knowledgeable* group. To interpret this tentative finding, cognitive and motivational characteristics should be taken into account (Kim et al., 2016). First, our results indicate that *motivated* pre-service teachers perceived the observation of the video clips and accompanying assessment tasks as less cognitively demanding than the other two profiles, as they reported the lowest perceived extraneous and intrinsic cognitive load. In other words, the *motivated* profile perceived the task difficulty as lower than the other two profiles. Comparing this to the *knowledgeable* profile, which tended to perceive a more extraneous and intrinsic load combined with a high germane load (Sweller et al., 2019), this again highlights that *motivated* learners may have applied learning activities in a less intensive way than the *knowledgeable* profile, possibly due to their high success expectancy. Nevertheless, the group of *motivated* learners also navigated the simulation successfully, indicating some kind of compensation for lower pre-knowledge with higher motivation.

Thus far, the advantages or disadvantages of particularly high expectancies and motivation for learning have been discussed, and mixed findings have been reported (Praetorius et al., 2016; Wigfield & Eccles, 2000). For example, longitudinal survey analyses in higher education have indicated higher learning gains for learners who tend to overestimate their abilities (Dupeyrat et al., 2011); however, most studies to date refer to learning gains of knowledge rather than of performance, such as assessment skills (Praetorius et al., 2016). Thus, further research is needed on the various learner characteristic profiles and their capacities for further learning and the various outcome measures.

### Limitations

When interpreting the results, the following methodological issues need to be considered. First, our sample size was rather small in comparison to other LPAs (Tein et al., 2013); however, the three profiles identified for this sample showed good conceptual interpretability and good fit indices with the data. Regarding the sample, one should also consider that the study was conducted at different universities to eliminate effects that could be related to a specific university and corresponding teacher education programs. For this reason, and due to the COVID-19 pandemic, the study was administered completely online, which might have led to a decreased control of data quality; however, in our data cleaning and analysis, we found no reason to doubt their quality. The voluntary nature of participation in the study may also have led to selection effects; however, the data did not indicate any specific bias within the sample.

Second, the results were surveyed in a video-based simulation focusing on pre-service teachers’ assessment skills regarding simulated students’ mathematical proof and argumentation skills. Thus, the study and its results are bound to the format of video-based simulations and a special domain-specific facet of assessment skills. To generalize our results, further studies need to be conducted concerning (1) other video-based simulations and (2) other facets of assessment skills.

Third, the operationalization of assessment skills was limited. The assessment process was measured by the number of words written and the number of videos watched; while superficial, these measures can be evaluated automatically and may thus be of particular interest when considering making the simulation adaptive. Further research is needed to generalize our findings on the possible underestimation of the assessment process by the *motivated* and *potentially struggling* profiles. Moreover, judgment quality has been primarily analyzed with the participants’ judgment accuracy, which is in line with other research (Urhahne & Wijnia, 2021); however, other aspects, such as judgment efficiency, are also relevant and should be considered in further research (Heitzmann et al., 2019).

## Conclusion

The aim of this study was to investigate the role of individual learner characteristics in situative learning experiences and the outcomes of video-based simulations as educational technology tools for initial teacher education. To advance the development and usage of simulations in a complex and practice-oriented field like teacher education, especially regarding the individualization of learning, a person-centered approach was applied, and three different learner characteristic profiles were identified. The results emphasize the role of individualization as they show that—depending on the individual learner characteristics—learners navigate differently through simulations, which in turn affects learning experiences and outcomes. The identification of learner profiles is considered a promising approach to identify particular learner needs to navigate simulations successfully. Thus, future research may consider the existence of learner profiles and their interactions with the simulation to identify and to investigate possible needs. Based on these needs, future research should consider adapting simulations by implementing targeted scaffolds for particular learner profiles.

## Data Availability

The datasets presented in this article are not readily available because they will be uploaded to a publicly accessible repository as part of the dataset of the DFG research group COSIMA (FOR2385). Requests to access the datasets should be directed to the corresponding author.

## Footnotes

1 Self-regulation does not only include motivational-affective but also cognitive and metacognitive aspects; however, as we focused primarily on its motivational-affective aspects, self-regulation is treated as a motivational-affective learner characteristic.

2 The gender imbalance in this study mirrors the current gender imbalance in the teaching profession in Germany (see for example OECD, 2019).
